# Birth Defects and Mothers’ Proximity to Natural Gas Development: Is There a Connection?

**DOI:** 10.1289/ehp.122-A109

**Published:** 2014-04-01

**Authors:** Lindsey Konkel

**Affiliations:** Lindsey Konkel is a Worcester, MA–based journalist who reports on science, health, and the environment. She is an editor for *Environmental Health News* and *The Daily Climate*.

More than 15 million Americans are now estimated to live within one mile of a natural gas well drilled since 2000.^1^ Research has demonstrated that natural gas development results in the emission of pollutants that include suspected developmental toxicants, such as benzene, toluene, and xylenes,^2^ although few studies have investigated the public health impact of these emissions. In this issue of *EHP*, researchers report preliminary evidence of an association between two birth defects and a mother’s residential proximity to natural gas wells at the time of birth.^3^

“Studies like this underscore the need for more representative and comprehensive research on workers and communities to understand their exposures and potential health risks,” says Aubrey Miller, a senior medical advisor at the National Institute of Environmental Health Sciences.

Researchers led by Lisa McKenzie at the Colorado School of Public Health estimated exposure to natural gas development for nearly 125,000 Colorado women. They used an “inverse distance weighting” method in which they determined the density of natural gas wells within a 10-mile radius of each mother’s home at the time she gave birth, with greater weighting for wells nearer the home. Then they compared proximity to gas wells between mothers who had adverse birth outcomes—including three types of birth defects, preterm birth, and term low birth weight—and those who did not.

**Figure:**
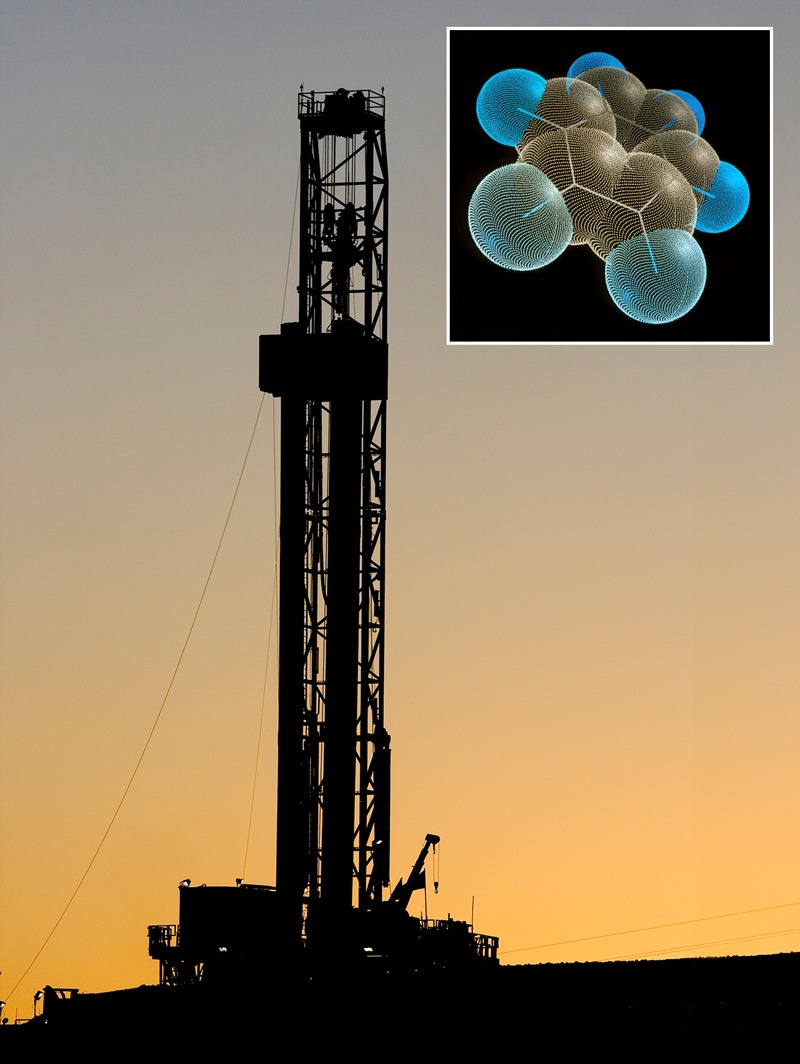
Future studies should assess exposure to benzene (inset) among people living near gas wells. Well: © Steve Starr/Corbis; molecule: © Alfred Pasieka/Science Source

Among 59 cases of neural tube defects, prevalence was twice as high among babies of mothers in the highest exposure group—a group of 19 women with more than 125 wells per mile within a 10-mile radius of the home—compared with babies of unexposed mothers. However, there was no evidence that neural tube defects were increased among the 13 babies of mothers classified as having low or medium exposure.

Among 1,823 cases of congenital heart defects, prevalence was 30% higher among babies of mothers in the highest exposure group, compared with babies born to unexposed mothers. In contrast to neural tube defects, however, the likelihood of a congenital heart defect increased steadily with increasing exposure. Congenital heart defects are the most common type of birth defect, affecting about 8 of every 1,000 newborns in the United States.^4^

There was no association observed between proximity to natural gas development and having babies with an oral cleft. In addition, babies whose mothers experienced the highest exposures to natural gas wells were slightly less likely to be born prematurely or at a low birth weight. However, the association between proximity to gas wells and improving birth weights diminished when the researchers accounted for elevation. “Higher elevations are associated with lower birth weights, while most natural gas drilling in Colorado occurs at low elevations,” McKenzie explains.

Although low levels of maternal folic acid—a B vitamin found in green leafy vegetables—is an established risk factor for neural tube defects,^5^ little is known about other environmental factors that may contribute to these birth defects. Two previous studies suggested that maternal exposure to benzene could increase congenital heart defects and neural tube defects.^6^^,^^7^ Another study reported associations between several birth defects in California and increased ambient concentrations of carbon monoxide and ozone.^8^

“The study certainly raises legitimate concerns, given that there are plausible mechanisms for many of the chemicals that are used in natural gas development,” says Kenneth Spaeth, medical director of the Occupational and Environmental Medicine Center at North Shore University Hospital inNew York, who was not involved in the study.

The authors suggest that exposure to benzene from the wells is one such plausible explanation for their findings. However, they did not identify or measure specific pollutants that may have been present at natural gas wells or specific activities occurring at the sites, so it is not possible to know which chemicals and/or other environmental stressors—if any—explain the associations. It’s also possible that some other risk factor not associated with wells, such as mothers’ folic acid consumption or level of prenatal care, could have influenced the results.

“Our findings are far from representing a causal effect,” says McKenzie. Her plans for future research include interviews with study mothers to get more information about their pregnancy and place of residence during the first trimester, a critical period for birth defect formation. She also plans to obtain more detailed information on specific activities taking place at well sites during the first trimester.
